# SYTO-13, a Viability Marker as a New Tool to Monitor *In Vitro* Pharmacodynamic Parameters of Anti-*Pneumocystis* Drugs

**DOI:** 10.1371/journal.pone.0130358

**Published:** 2015-06-23

**Authors:** Annie Standaert-Vitse, Cécile-Marie Aliouat-Denis, Anna Martinez, Sara Khalife, Muriel Pottier, Nausicaa Gantois, Eduardo Dei-Cas, El Moukhtar Aliouat

**Affiliations:** 1 Biology & Diversity of Emerging Eukaryotic Pathogens (BDEEP), Center for Infection and Immunity of Lille (CIIL), INSERM U1019, CNRS UMR 8204, University of Lille, Pasteur Institute of Lille, Lille, France; 2 RNA Processing Research Group, Biomedical Research Institute, National Institute of Advanced Industrial Science and Technology (AIST), Ibaraki, Japan; 3 Centre AZM pour la Recherche en Biotechnologie et ses Applications, Laboratoire Microbiologie, Santé et Environnement, Université Libanaise, Tripoli, Lebanon; 4 CHRU Lille, Biology & Pathology Center, Parasitology-Mycology, Lille, France; Duke University, UNITED STATES

## Abstract

While *Pneumocystis* pneumonia (PcP) still impacts the AIDS patients, it has a growing importance in immunosuppressed HIV-negative patients. To determine the anti-*Pneumocystis* therapeutic efficacy of new compounds, animal and *in vitro* models have been developed. Indeed, well-designed mouse or rat experimental models of pneumocystosis can be used to describe the *in vivo* anti-*Pneumocystis* activity of new drugs. *In vitro* models, which enable the screening of a large panel of new molecules, have been developed using axenic cultures or co-culture with feeder cells; but no universally accepted standard method is currently available to evaluate anti-*Pneumocystis* molecules *in vitro*. Thus, we chose to explore the use of the SYTO-13 dye, as a new indicator of *Pneumocystis* viability. In the present work, we established the experimental conditions to define the *in vitro* pharmacodynamic parameters (EC50, Emax) of marketed compounds (trimethoprim/sulfamethoxazole, pentamidine, atovaquone) in order to specifically measure the intrinsic activity of these anti-*P*. *carinii* molecules using the SYTO-13 dye for the first time. Co-labelling the fungal organisms with anti-*P*. *carinii* specific antibodies enabled the measurement of viability of *Pneumocystis* organisms while excluding host debris from the analysis. Moreover, contrary to microscopic observation, large numbers of fungal cells can be analyzed by flow cytometry, thus increasing statistical significance and avoiding misreading during fastidious quantitation of stained organisms. In conclusion, the SYTO-13 dye allowed us to show a reproducible dose/effect relationship for the tested anti-*Pneumocystis* drugs.

## Introduction

*Pneumocystis* pneumonia (PcP) still has a high impact on AIDS patients from developed or developing world areas because of newly diagnosed HIV infections, non adherence or limited access to either highly active antiretroviral therapy (HAART) or PcP chemoprophylaxis [1]or high contribution to AIDS mortality [2]. In HIV-negative patients submitted to immunosuppressive treatments for malignancies, auto-immune diseases or organ transplantation, PcP is the cause of pneumonia in 10%-40% of these patients, with high mortality rates [3, 4]. In addition, *Pneumocystis* organisms were detected relatively frequently in neonates or small children [5, 6], pregnant women [7] and patients with chronic underlying diseases [8–10]. Indeed, in the latter population, *Pneumocystis* organisms have been considered as co-morbidity factors worsening the prognosis [3, 5, 11, 12]. Particularly, in patients with chronic obstructive pulmonary disease (COPD), a colonization of the lungs by *P*. *jirovecii* is associated with a more progressive and severe disease [12, 13, 14]. In clinical practice, the therapeutic choices are limited due to the lack of therapeutic alternatives to the well-known trimethoprim-sulfamethoxazole (TMP/SMX) drug combination and warrant the search for more effective and less toxic agents. Moreover, some recent reports have suggested the emergence of *P*. *jirovecii* resistance to sulpha drugs [15, 16].

The therapeutic efficacy of new compounds against *Pneumocystis* micromycetes, is usually determined using well-defined mouse or rat experimental models of pneumocystosis [17–21] or/and *in vitro* models.

Since no continuous culture system supporting the replication of the organism is available, *in vitro* models, which enable the screening of a large panel of new molecules, have been developed using axenic cultures or co-cultures with feeder cells [22–24]. However, no universally accepted standard method is currently available to evaluate anti-*Pneumocystis* molecules *in vitro*. The main reasons for this situation are (i) the microscopic observation of *Pneumocystis* is time consuming and requires a great expertise [25, 26] and (ii) the assessment of *Pneumocystis* viability with high confidence and reproducibility is lacking [27]. Many methods have been described such as the incorporation of classical vital stains or fluorescent indicator compounds [26, 28, 29], the ATP bioluminescent assay [27, 30], the uptake of radiolabelled methionine, uracil or thymidine [31], and the RT-PCR [32, 33]. However, none of these overcomes the host cell debris interferences and none has permitted to monitor the concentration-effect relationships (i.e. pharmacodynamics parameters) of anti-*Pneumocystis* compounds. As an alternative method, we evaluated the use of SYTO-13, a member of the class of SYTO dyes, as a new indicator of *Pneumocystis* viability. The SYTO dyes, among which is the most popular SYTO-13 [34], are cell-permeant nucleic acid stains that permit myriad of applications such as the discrimination between live/dead eukaryotic or prokaryotic cells [35–37], the detection of apoptosis [38, 39], or germinated bacterial endospores [40].

The aim of the present work was to establish the *in vitro* experimental conditions allowing the definition of the *in vitro* pharmacodynamic parameters of three marketed compounds (TMP/SMX, pentamidine, atovaquone). For the first time, the SYTO-13 dye enabled to specifically characterize the intrinsic anti-*Pneumocystis* activities of these molecules and to show a reproducible dose/effect relationship, while being more reproducible and avoiding laborious microscopic observations.

## Results

### Labelling of *P*. *carinii* organisms with the SYTO-13 live-cell nucleic acid stain

**Fig 1:**
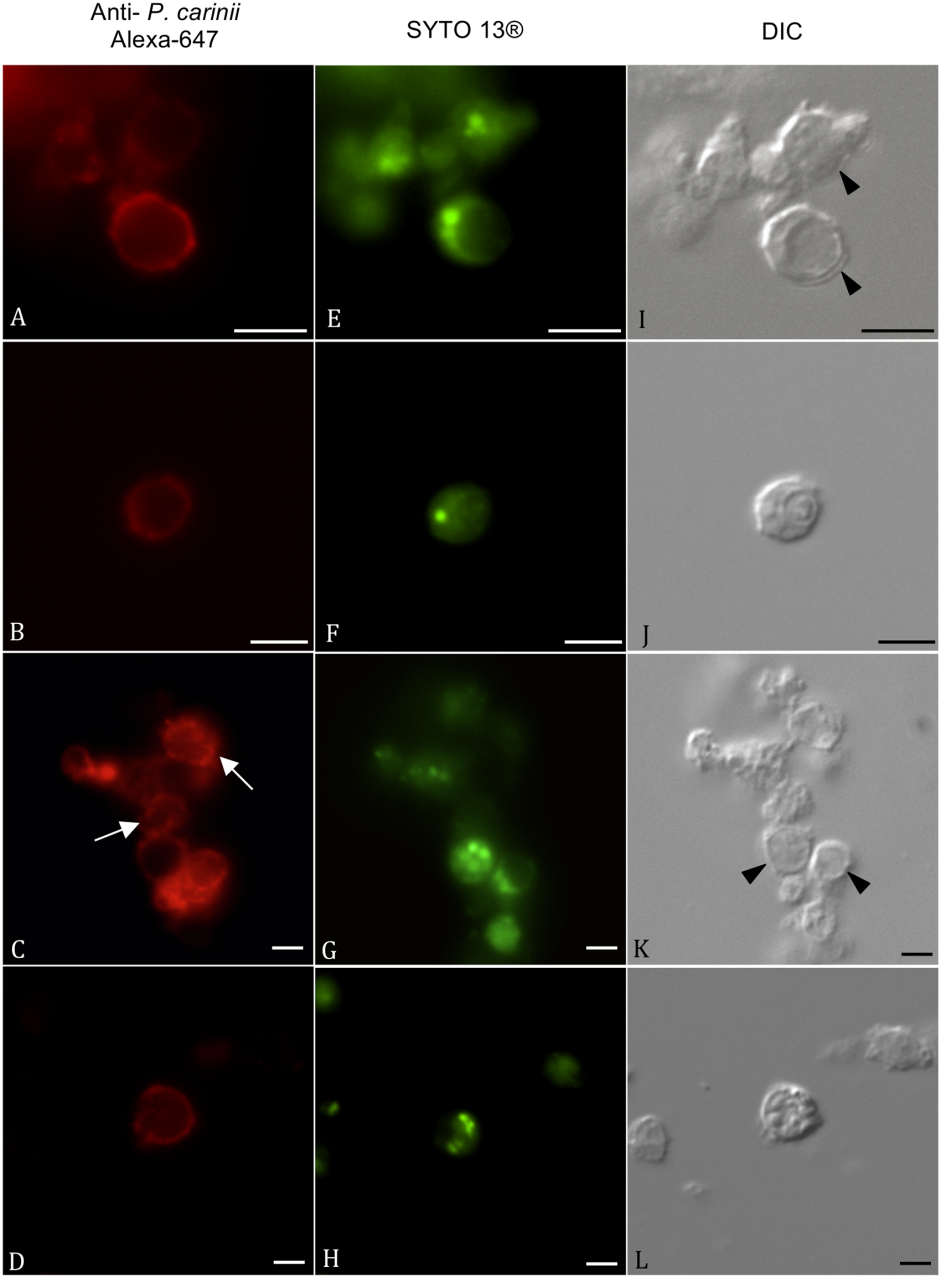
SYTO-13 labelling of *P*. *carinii* trophic and cystic forms. All *P*. *carinii* life cycle stages were stained in red (panels A–D) with a home-made anti-*Pneumocystis* polyclonal antibody, recognized by an Alexa-647-conjugated secondary antibody. SYTO-13 nuclear staining of viable *P*. *carinii* is displayed for corresponding fields (panels E–H). Differential Interference Contrast (DIC) pictures of corresponding fields are also shown (I–L). Panels (B, F, J) and (D, H, L), show an isolated cystic form with one or several labelled nuclei. In the other panels, cystic forms (arrowheads) and trophic forms are well visible. The white arrows show non-viable *Pneumocystis* organisms stained in red but did not display any nuclear green fluorescence (Panels C, G, K). Bar = 5 μm.

*P*. *carinii* was stained using a specific anti-*Pneumocystis* polyclonal antibody and a goat anti-rat IgG conjugated to Alexa-647 (Fig 1). *Pneumocystis* trophic and cystic forms are both labelled in red. The host lung debris were not labelled by the polyclonal antibody.

SYTO-13 is a sensitive DNA stain broadly used for viability studies. SYTO-13 dye easily penetrates most cell types and undergoes dramatic fluorescence enhancement upon binding to nucleic acids. Thus, when *P*. *carinii* was incubated with SYTO-13, the nuclei from living cells were stained in green (Fig 1). Cells that were stained in red but did not display any nuclear green fluorescence were considered as non-viable *P*. *carinii* organisms (Fig 1C, 1G and 1K).

### *P*. *carinii* viability assessment

**Fig 2:**
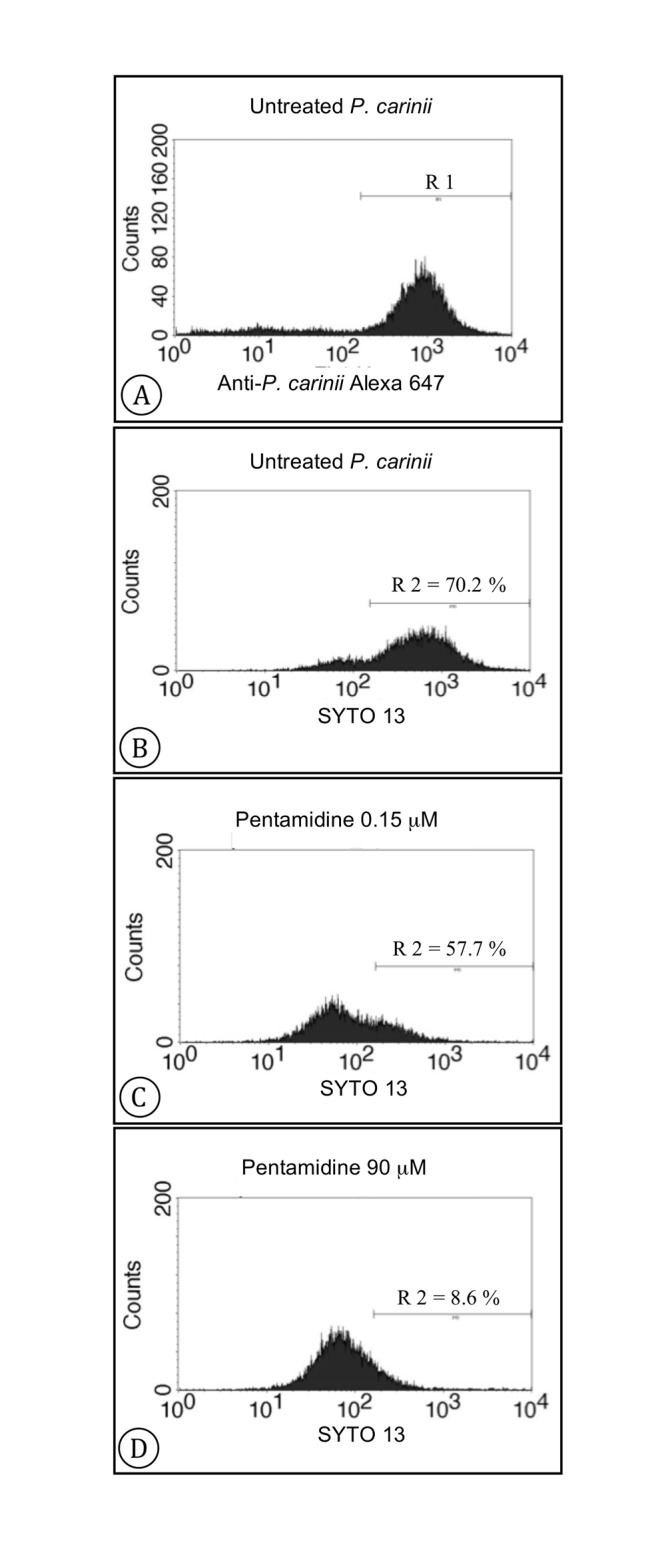
Viability assessment of *P*. *carinii* using SYTO-13 by flow cytometry analysis. The data are displayed as histograms displaying 10,000 *P*. *carinii* collected events. The axes represent the relative number of cells (y axis) and the cell-associated fluorescence intensity on a logarithmic scale (x axis). Panel A shows the gated (R1) *P*. *carinii* population labelled with a rat specific polyclonal antibody and a goat anti-rat IgG antibody conjugated to Alexa-647 (FL4 channel). The intensity of SYTO-13 live-cell nucleic acid staining is analyzed within the gated *P*. *carinii* population (FL1 channel) for *P*. *carinii* organisms cultured during 4 days without (panel B), with 0.15 μM (panel C) or 90 μM (panel D) of pentamidine. The gated population R2 represents the viable *Pneumocystis* cells. The percentages of viability are indicated for each histogram. The presented histograms are representative of one experiment.

To specifically measure the viability of *Pneumocystis* organisms, a first step consisted in selecting the labelled *P*. *carinii* population using flow cytometry (Fig 2). Thus, the gated population R1 (Fig 2A) allowed the isolation of *P*. *carinii* organisms from the host pulmonary cell debris. Then, the SYTO-13 fluorescence signals were analyzed within R1. Fig 2B represents the viable population (R2 gate) of untreated *P*. *carinii* after 4 days of culture in DMEM with 10% FCS. The percentage of viability reached 72.00 ± 7.02% (n = 8) for untreated cultured *Pneumocystis* organisms. When *P*. *carinii* cells were incubated with increasing concentrations of anti-*Pneumocystis* drugs, the number of viable cells (R2) decreased gradually until hardly no viable *P*. *carinii* organism was detected: as an example, 56.70 ± 1.56% (n = 3) and 8.60 ± 0.04% (n = 3) of viable *P*. *carinii* organisms for 0.15 μM and 90 μM of pentamidine, respectively (Fig 2C and 2D).

### Pharmacodynamic parameters

**Fig 3:**
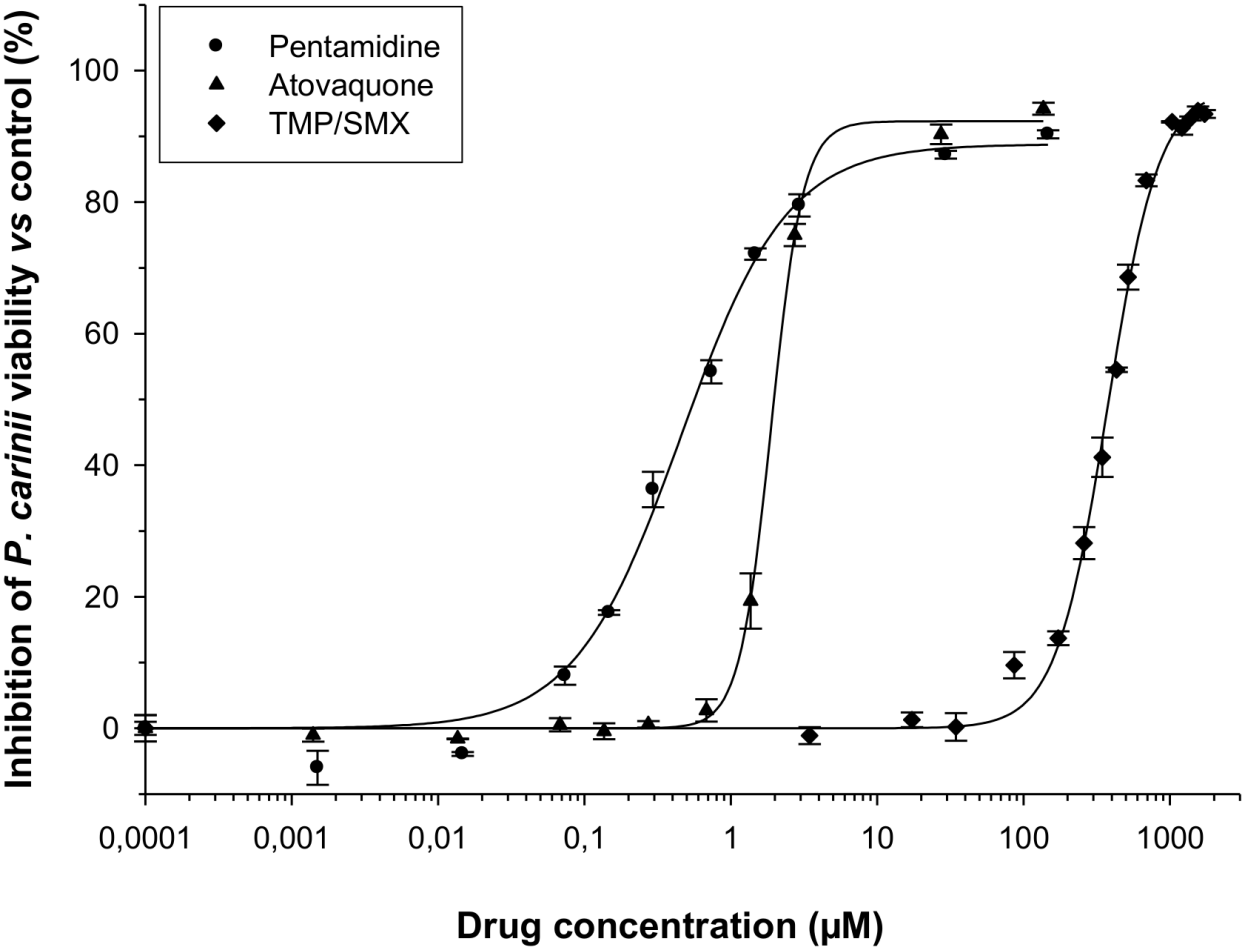
Relationships between drug concentrations and *P*. *carinii* viability inhibition of pentamidine, atovaquone and trimethoprim-sulfamethoxazole (TMP/SMX). Values of *P*. *carinii* viability inhibition are calculated from the SYTO-13 live-cell staining assay. To calculate the percentage of viability inhibition in relation with drug concentrations, one drug-free control was included in each assay. All susceptibility assays were set up in triplicate.

The inhibition of *P*. *carinii* viability in the presence of anti-*Pneumocystis* drugs was compared to drug-free control using the SYTO-13 assay (Fig 3). The concentration-response curves were plotted after 4 days of incubation of *P*. *carinii* organisms with pentamidine, atovaquone or TMP/SMX. The reduction in the number of viable microorganisms was gradual and dependent of drug concentration. Data were integrated using the Hill equation (sigmoid Emax model) to calculate the pharmacodynamic parameters of each tested drug (Table 1) as indicated in Materials and Methods.

**Table 1:** *In vitro* pharmacodynamic parameters of pentamidine, atovaquone and trimethoprim-sulfamethoxazole (TMP/SMX) calculated using the SYTO-13 assay.

Drug	EC_50_ (μM)	Slope
Pentamidine	0.45 ± 0.05	1.20 ± 0.12
Atovaquone	1.89 ± 0.04	4.00 ± 0.20
TMP/SMX	373.8/2089 ± 12.07 /72.73	2.46 ± 0.20

TMP/SMX demonstrated the lowest intrinsic potency with an EC50 of 373.8/2089 μM (Table 1). In comparison, pentamidine showed a maximum killing rate of *P*. *carinii* with an EC50 value of 0.45 μM and atovaquone, with an EC50 value of 1.89 μM. In terms of efficacy, pentamidine and atovaquone reached at least 90% of inhibition at a concentration of 145 μM. Higher concentrations of TMP/SMX (1/6 mM) were needed to achieve 90% of inhibition. Finally, our data showed that the potency of atovaquone and TMP/SMX were very sensitive to variations of drug concentration, as attested by the important steepness of the curves (4.00 and 2.46, respectively, Table 1).

## Discussion

The availability of a robust and consistent method to assess the intrinsic activity of drugs is a key step in the development of new anti-*Pneumocystis* treatments. A pharmacodynamic model was previously developed to that purpose [20, 41]. Thus, the *in vitro* drug activities were determined using the broth microdilution technique, comparing the total number of microorganisms in treated and drug-free cultures as monitored after Giemsa staining. The *in vitro* maximum effect (Emax, efficacy), the drug concentrations for which 50% of Emax is obtained (EC50, potency), and the slope of the dose-response curves were then calculated by the Hill equation (sigmoid Emax model). However, two drawbacks arise from this methodology: (i) the microscopic quantitation of *Pneumocystis* organisms is time-consuming and requires a great expertise; (ii) the Giemsa staining does not enable to distinguish viable from non-viable *Pneumocystis* organisms although assessing the viability remains a key feature to evaluate antimicrobial activity accurately.

Most viability assays using the light microscope are not really applicable to *Pneumocystis*. Indeed, the interpretation of results acquired with most staining methods is often inconclusive. Only a double-staining fluorogenic method [28, 29] was considered to be reliable to evaluate the viability of *Pneumocystis* fungal cells. This method employs a carboxyfluorescein diacetate labelling, which positively stains live parasites in fluorescent green, combined with propidium iodide, that intercalates into double-stranded nucleic acids, and stains only dead parasites in fluorescent red. Other authors used propidium iodide alone to assess the viability of *P*. *carinii* by flow cytometry analysis [26]. Viability was also evaluated by monitoring metabolic activities such as the *de novo* synthesis of folates [42], the incorporation of para-aminobenzoic acid (pABA) [43], or the uptake of methionine, uracil, thymidine [31] or dimethyaminostyrylmethylpyrimidinium-iodide (DASPMI) [44]. Moreover, some authors used detection of Heat Shock Protein 70 transcripts by RT-PCR to monitor the viability of *P*. *jirovecii* in bronchoalveolar lavage (BAL) fluids from patients developing PcP [32, 33] or in air samples collected from the environment of PcP patients [33]. However, the concentration-effect relationship (*i*.*e*. pharmacodynamic parameters) of anti-*Pneumocystis* compounds has not been characterized by these methodologies. Moreover, the viability assays based on microscopic observations are limited because of the low numbers of analyzed cells and potential interferences due to the host cell debris.

In the present work, SYTO-13, a member of the class of SYTO dyes, is proposed as a new tool to monitor *Pneumocystis* viability. The SYTO live-cell nucleic acid stains are sensitive DNA stains that are broadly used for live-cell studies [35–37]. The SYTO dyes readily penetrate most cell types and undergo dramatic fluorescence enhancement upon binding to nucleic acids as we have shown it in our experiments (Figs 1 and 2). The SYTO-13 dye has proven valuable in various fields of research applications such as (i) live/dead discrimination in eukaryotic or prokaryotic cells [35–37], (ii) detection of apoptosis [38, 39] and (iii) quantification of bacterial endospore germination [40].

In our hands, SYTO-13 also revealed to be an excellent dye to assess the effect of anti-*P*. *carinii* drugs. Indeed, the results concerning the response to standard anti-*Pneumocystis* drugs obtained in our pharmacodynamic in vitro models using SYTO-13 were consistent with previous in vitro efficacy studies [20, 41]. However, the EC50 values calculated after Giemsa staining (using the same procedure and *Pneumocystis* strain detailed in references [20, 41]) are 2- to 6-fold higher than those obtained with SYTO-13 highlighting the difficulty to distinguish viable *P*. *carinii* organisms by microscopic observations. In other words, there is a tendency to over-estimate the amount of dead *P*. *carinii* after Giemsa staining.

SYTO-13 allowed the accurate follow-up of the shift of *P*. *carinii* organisms from the viable to the dead compartment upon increase of drug concentrations (Fig 2B, 2C and 2D). Thus, it became feasible to calculate the percentage of viability inhibition in relation to untreated *P*. *carinii* control. By using anti-*P*. *carinii* specific antibodies, we developed a specific and sensitive tool to assess the viability of *P*. *carinii* organisms in the presence of marketed compounds, while selecting fungal organisms against host cell debris. Moreover, a large number of fungal cells was analyzed by flow cytometry leading to consistent and objective results, while avoiding misreading or false interpretation that often occurs when observing Giemsa-stained and rather small (2–8 μm in diameter) *P*. *carinii* organisms under the microscope.

In conclusion, the SYTO-13 staining assay combined with a flow cytometry analysis appears as a simple and reliable approach for the measurement of *Pneumocystis* viability. We have also demonstrated that SYTO-13 is compatible with the application of the pharmacodynamic Emax model, enabling to show a reproducible dose/effect relationship for the tested anti-*Pneumocystis* marketed drugs. Finally, it could be interesting to investigate the use of SYTO-13 to measure the viability of *P*. *jirovecii* collected from patients with PcP and subsequently cultured in the presence of drugs for a short period of time in order, for example, to explore the potency of drug treatment.

## Materials and Methods

### Drugs

Trimethoprim (TMP), pentamidine and atovaquone (Sigma-Aldrich, France) were dissolved in 100% dimethyl sulfoxide (DMSO, Sigma Aldrich, St. Louis, Missouri) to produce a 90 mM stock solution. Sulfamethoxazole (SMX, Sigma-Aldrich, France) was also dissolved in DMSO but at a concentration of 600 mM. TMP and SMX solutions were mixed appropriately to obtain a final combination of 1:5. Finally, the drug stock solutions were diluted in Dulbecco's Modified Eagle's Medium (DMEM, BioWhittaker Europe, Verviers, Belgium) supplemented with 10% heat-inactivated fetal calf serum (FCS, GIBCO BRL, Life Technologies Inc., France) to produce the required drug concentrations. Compound solutions were prepared immediately before use.

### Ethics statement

All animal experiments were performed according to the directive 2010/63/EEC on the Protection of Animals Used for Experimental and Other Scientific Purposes. The animal work also complied with the French law (nu 2012–10 dated 05/01/2012 and 2013–118 dated 01/02/2013). All experimental protocols involving animals were carried out by qualified personnel. The animal house (accreditation number: A59107, agreement number: B 59–350009) was placed under the direct control of the director of the Pasteur Institute of Lille who is the ‘‘designated responsible person” under French law. The study has been approved by the Ethical Committee for experiments on animals of the Nord-Pas-de-Calais region (approval number CEEA 022011).

### Source of *P*. *carinii* organisms

Athymic *Pneumocystis*-free Lou nu/nu rats (Pasteur Institute Lille, France) were used as source of *Pneumocystis carinii* organisms for all experiments [45]. Ten-week-old female nude rats were administered dexamethasone (Merck Sharp & Dohme Chibret, Paris, France) for two weeks in the drinking water (1 mg/L). Then, rats were inoculated with 10^7^ of cryopreserved parasites using a non-surgical endotracheal method [18]. Dexamethasone treatment was maintained until the end of the experiment. Six to 8 weeks post-inoculation (p.i.), rats were highly infected by *P*. *carinii*, without secondary fungal or bacterial infection. Animals were housed in HEPA-filtered air isolators (Flufrance, Wissous, France) and were allowed sterile irradiated food (Scientific Animal Food & Engineering, SAFE, Augy, France) and sterile water *ad libitum*.

### Extraction, purification and quantitation of *P*. *carinii* organisms

Six to 8 weeks following inoculation, rats were euthanatized and parasite extraction was performed as previously described [20]. Briefly, parasites were extracted in DMEM (BioWhittaker, France) by agitation of lung pieces with a magnetic stirrer. The resulting homogenate was poured successively through gauze, 250 and 63 μm stainless steels filters. After centrifugation, the pellet was suspended in an haemolytic buffered solution. *P*. *carinii* organisms were collected by centrifugation and then purified on a polysucrose gradient (Histopaque-1077, Sigma-Aldrich, France). Blood and Sabouraud dextrose agar (Difco, France) media were inoculated with purified parasites to check for the absence of contaminating pathogens. *P*. *carinii* was quantitated on air dried smears stained with RAL-555 (Réactifs RAL, Martillac, France), a rapid panoptic methanol-Giemsa-like staining, which stains trophic forms, sporocytes and cysts of *P*. *carinii* (20). *P*. *carinii* was then cryopreserved by placing parasites in FCS with 10% DMSO at -80°C in a Nalgene 1°C cryo-freezing container (cooling rate: about 1°C/min) for 4 hours [46]. The parasite samples were then stored in liquid nitrogen. Cryopreserved *P*. *carinii* were used for *in vitro* anti-microbial studies.

### Axenic *in vitro* culture of *P*. *carinii*

In order to determine *in vitro* drug susceptibility of *P*. *carinii*, axenic cultures of the organism were performed as follows [20]. All the experiments were carried out in 24-well plates with a final volume of 2 mL of DMEM supplemented with 10% FCS containing a final inoculum of 10^6^ organisms per mL. Plates with organisms were incubated for 4 days in an atmosphere of 5% CO_2_ at 37°C. Then, the total volume of each well was removed, centrifuged for 10 min at 2,900 *× g* and the pellet was suspended with 100 μL of phosphate buffer solution (PBS) Dulbecco (Sigma Chemical Co.).

### *In vitro* susceptibility studies

*In vitro* susceptibility studies were performed using the twofold broth microdilution technique. Final drug concentrations ranged from 150 μM to 1.5 nM for pentamidine and atovaquone. TMP/SMX combination was tested from 1.7/10 mM to 3.5/20 μM. Plates were incubated during 4 days in an atmosphere of 5% CO_2_ at 37°C. Viability of *Pneumocystis* organisms incubated in such culture conditions, but without any drugs, was assessed using the SYTO-13 assay and was set to 100% of viability. Such a control is named the drug-free control. All susceptibility assays were set up in triplicate.

### *Pneumocystis* labelling

An immunofluorescence assay (IFA), using a specific anti-*Pneumocystis* polyclonal antibody, was performed directly in each 100μL-parasite culture suspension, prior analysis by flow cytometry. We used a specific anti-*Pneumocystis* polyclonal antibody made in our laboratory as follows: *P*. *carinii* endotracheally-inoculated Sprague Dawley rats (Harlan, France) were immunosuppressed for 10 weeks with dexamethasone (2 mg/L in the drinking water). The infected animals produced high *Pneumocystis* loads. Then, animals received half of the dexamethasone dose for one week before stopping the immunosuppression treatment. After 8 to 10 weeks of recovery, the antibody titer was determined in the collected serum. The *P*. *carinii* trophic and cystic forms were labelled with high intensity by the polyclonal antibody as checked by IFA.

A goat anti-rat IgG (H+L) conjugated to Alexa-647 (Invitrogen Life Sciences, Cergy Pontoise, France) was used as secondary antibody. The optimal dilutions used for the polyclonal antibody and the secondary antibody were 1:100 and 1:50, respectively. The organisms were washed once with PBS Dulbecco (10 min at 2,900 *× g*) between each antibody incubation steps. Antibodies were incubated 30 min at 37°C.

### *Pneumocystis* viability using SYTO-13 live-cell nucleic acid stain

After *Pneumocystis* labelling with the specific polyclonal antibody, each 100μL-parasite culture suspension was incubated at room temperature with 1 mL of SYTO-13 (Molecular Probes Europe BV, Leiden, The Netherlands). Different concentrations of SYTO-13 (1 to 20 μM) and different incubation times (10 to 60 min) have been tested. The optimal concentration to label *Pneumocystis* organisms were found to be 2.5 μM of SYTO-13 in PBS Dulbecco with an incubation time of 20 min.

### Flow cytometry analysis

Immunostained *P*. *carinii* organisms, that were incubated with SYTO-13, were analyzed using the FACScalibur 2 flow cytometer (Becton Dickinson) driven by the BD CellQuest software (version 0.3.df6b, Becton Dickinson). The cytometer is equipped with an air-cooled blue laser providing 15 mW at 488 nm and the standard filter setup. All parameters were collected as logarithmic signals. Green fluorescence (SYTO-13 staining) was collected in the FL1 channel (530 ± 30 nm) whereas red fluorescence (Alexa-647 goat anti-rat IgG; *P*. *carinii* staining) was collected in the FL4 channel (661 ± 16 nm). The SYTO-13-stained *P*. *carinii* organisms were numbered within the gate selecting for the anti-*P*. *carinii* polyclonal antibody positive staining. The viable *P*. *carinii* are gated (R2, Fig 2) inside the cell population with high green fluorescence and the percentage of viability is then calculated in comparison with all events. The *in vitro* activity of tested compounds against *P*. *carinii* was expressed as a percentage of viability inhibition defined as the total number of SYTO-13 positive parasites for a given drug concentration in comparison with the number of untreated parasites.

### Determination of pharmacodynamic parameters

Once the percentages of inhibition of *P*. *carinii* viability were calculated, the Hill equation was applied to establish the relationship existing between the concentration and the inhibitory effect of a given drug as follows [47]: E_R_ = (E_R,max_ x C^S^) / [(EC_50_)^S^ + C^S^]. ***E***_***R***_ is the effect of each drug concentration on the percentage of inhibition estimated from experimental results (***C***); ***S*** is a parameter reflecting the steepness of the concentration-effect relationship curve; ***EC***_***50***_ is the concentration of the compound at which 50% of the maximum effect (***E***_***R*,*max***_) is reached.

The parameters of this pharmacodynamic model were calculated by nonlinear least-square regression techniques using a commercial software (WinNonlin).
